# Role of iron in the reduction of anemia among women of reproductive age in low-middle income countries: insights from systematic review and meta-analysis

**DOI:** 10.1186/s12905-023-02291-6

**Published:** 2023-04-17

**Authors:** Sumera Aziz Ali, Shama Razzaq, Savera Aziz, Ahreen Allana, Arzina Aziz Ali, Shahla Naeem, Nayab Khowaja, Fazal Ur Rehman

**Affiliations:** 1 Department of Epidemiology, Columbia University, New-York, USA; 2 Divison of Environment and Sustainability, The Hong Kong University of Science and Technology, Hong Kong, People’s Republic of China; 3 Faculty of Nursing, University of Alberta, Alberta, Canada; 4 Aga Khan University Hospital, Karachi, Pakistan; 5 CMH Institute of Medical Sciences, Bahawalpur, Pakistan; 6 Liaquat University of Medical and Health Sciences, Jamshoro, Pakistan

**Keywords:** Anemia, Iron therapy, Women of reproductive age, Systematic review, Meta-analysis, Low-middle income countries

## Abstract

**Background:**

Iron deficiency anemia is a common public health issue among women of reproductive age (WRA) because it can result in adverse maternal and birth outcomes. Although studies are undertaken to assess iron efficacy, some gaps and limitations in the existing literature need to be addressed. To fill the gaps, we conducted a systematic review and meta-analysis of randomized controlled trials (RCTs) assessing the role of iron in reducing anemia among WRA in low-middle-income countries (LMICs).

**Methods:**

A comprehensive search strategy was used to search Medline through PubMed, Embase, and Science Direct for RCTs published between 2000 and 2020. The primary outcome was the mean change in hemoglobin level. We used standardized mean differences and their respective 95% CI to estimate the pooled effect. We used I^2^ statistics and Egger’s test to assess heterogeneity and publication bias, respectively. This review was carried out in accordance with revised guidelines based on the Preferred Reporting Items for Systematic Review and Meta-analysis.

**Results:**

The findings showed that iron therapy improved hemoglobin and ferritin levels, though the results varied across studies. An overall pooled effect estimate for the role of iron therapy in improving the hemoglobin levels among WRA was -0.71 (95% CI: -1.27 to -0.14) (*p* = 0.008). Likewise, the overall pooled effect estimate for the role of iron therapy in improving the ferritin levels among WRA was -0.76 (95% CI: -1.56 to 0.04) (*p* = 0.04). The heterogeneity (I^2^) across included studies was found to be statistically significant for studies assessing hemoglobin (Q = 746.93, I^2^ = 97.59%, *p* = 0.000) and ferritin level (Q = 659.95, I^2^ = 97.88%, *p* = 0.000).

**Conclusion:**

Iron therapy in any form may reduce anemia’s burden and improve hemoglobin and ferritin levels, indicating improvement in iron-deficiency anemia. More evidence is required, however, to assess the morbidity associated with iron consumption, such as side effects, work performance, economic outcomes, mental health, and adherence to the intervention, with a particular focus on married but non-pregnant women planning a pregnancy in the near future.

**Trial registration:**

Registered with PROSPERO and ID is CRD42020185033.

**Supplementary Information:**

The online version contains supplementary material available at 10.1186/s12905-023-02291-6.

## Background

Iron deficiency anemia is a significant concern for women of reproductive age (WRA) because it can have adverse maternal and fetal outcomes [1]. Because of the severe consequences of anemia for both WRA and their offspring, the World Health Organization (WHO) has prioritized iron deficiency anemia on the global health agenda [2]. The existing literature reveals that anemic pregnant women are more likely to have miscarriages, intrauterine growth retardation, preterm births, stillbirths, babies born with low birth weight (LBW), and neonatal mortality [3–6]. Previous studies, for example, show that a 10 gm increase in hemoglobin levels results in a 30% decrease in maternal deaths, indicating a linear relationship between anemia and maternal mortality [7]. Similarly, anemia during pregnancy contributes to 25% of LBW, 44% of preterm births, and roughly a quarter of stillbirths and neonatal deaths in resource-limited settings [8]. A meta-analysis found that anemia during pregnancy increased the risk of premature birth [9]. Furthermore, according to a WHO multi-country survey, severe anemia doubles the risk of maternal death [10].

While anemia is prevalent among WRA in both high and low and middle-income countries (LMICs), the latter experience a higher burden of anemia [11, 12]. Anemia affects approximately 66.7% of WRA in LMICs, possibly due to a poor diet caused by food insecurity, parasitic infections, and decreased iron absorption [13, 14]. Anemia affects roughly half a billion women worldwide, with South-East Asia accounting for 42% of those affected, followed by Africa and the Eastern Mediterranean [15]. In South Asian and African countries, approximately 50% of the burden of anemia among WRA is due to iron deficiency [16, 17]. For example, the burden of anemia ranges between 50 to 90% across diverse states of India [18–20]. Similarly, anemia affects 34.5% to 56.8%, 63.1%, and more than a third of the WRA in Ethiopia, Uganda, and Bangladesh, respectively [21–24].

While the etiology of anemia is multifactorial, inadequate iron store as a result of poor nutrition has been identified as an important risk factor for anemia among WRA [25, 26]. Women of reproductive age are more likely to suffer from iron deficiency due to menstruation, and this risk increases during pregnancy due to increased metabolic demands of pregnancy and fetal growth [25, 26]. To address the high prevalence of anemia, the WHO proposes to distribute iron supplements to all WRA in regions with an anemia burden of more than 20% [27].

Even though several interventions have been shown to reduce the global burden of anemia by 12% between 1992 and 2011, such interventions did not benefit WRA living in LMICs [28]. In addition, numerous epidemiological studies, including randomized controlled trials (RCTs), are being conducted to evaluate the role of iron in reducing the burden of anemia among WRA in LMICs. The findings of these RCTs have been synthesized in a few systematic reviews and meta-analyses; however, these meta-analyses from the last ten years are not without gaps and limitations. For instance, a Cochrane review by Low Yuan et al. (2016) assessed the effect of iron therapy in reducing anemia among menstruating women [29]. This review only included non-pregnant women and excluded pregnant women with a higher burden of anemia during pregnancy and requiring special care to avoid the negative feto-maternal outcomes associated with iron deficiency anemia [29]. In contrast, another systematic review that was undertaken in 2015 only focused on pregnant women and excluded non-pregnant women [30]. Haider et al. (2013) conducted a meta-analysis to evaluate the effect of iron therapy, but the study results were largely based on observational cohort and quasi-experimental studies, which are not free of residual and unmeasured confounding [31]. Finally, rather than studying iron deficiency markers as an outcome, Pasricha et al. (2014) conducted a meta-analysis on iron therapy's effect on women's physical exercise [32].

As previously stated, the limitations of existing reviews limit the capacity of health professionals and decision-makers in LMICs to address the problem of iron deficiency in both pregnant and non-pregnant women. Given the existing gaps in the literature, a systematic review of randomized controlled trials (RCTs) was required to assess the effect of only iron therapy in reducing anemia, primarily among pregnant and non-pregnant women living in LMICs. Hence, we conducted a systematic review and meta-analysis of RCTs to assess the existing evidence on the efficacy of iron therapy in lowering the burden of anemia and improving iron deficiency markers among WRA in LMICs. This review will help health professionals, researchers, and decision-makers in LMICs make evidence-based decisions when prescribing iron therapy to WRA. As a result, the high prevalence of iron deficiency anemia among WRAs living in resource-limited settings will be addressed.

## Material and methods

### Searching strategy

The updated Preferred Reporting Items for Systematic Reviews and Meta-Analyses (PRISMA) guidelines were employed to undertake this review and meta-analysis for the qualitative and quantitative synthesis of literature [33]. We focused on reviewing the evidence for the efficacy of iron as a therapy or intervention to reduce the burden of anemia among WRA in LMICs. Using keywords and specific search terms such as Medical Subject Headings (PubMed) and Emtree, a systematic search of three major electronic databases, including Medline through PubMed, Embase, and Science Direct, was undertaken.

### Eligibility criteria

Before conducting a systematic review of the literature and meta-analysis of the studies, we defined eligibility criteria to include the relevant studies undertaken predominantly in LMICs. World Bank’s 2018 guidelines of country classification were used to define a country as an ‘LMIC.’ The eligibility of a study was contingent on being primarily an RCT assessing the efficacy of iron in decreasing anemia among women aged 15–49 years in LMICs and being published in the English language in a peer-reviewed scientific journal from January 2000 to December 2020. A form to screen the studies based on eligibility criteria is given in Table 1.

**Table 1:** Screening form to assess the eligibility of the potential research articles

Study Characteristics			Page/ Para/ Figure #
Type of Study (Interventional studies)	□ Observational study • □ Case–control • □ Cross-sectional • □ Cohort	□ Interventional study • □ Randomized controlled trial • □ Quasi-experimental study • □ Pre-post design	
	□ Qualitative study • Exploratory • Descriptive • Ethnography • other	□ Other design (specify):	
	*Does the study design meet the criteria for inclusion?* Yes □ No □ →Exclude Unclear □		
Study Participants (Studies involving women of reproductive age 15–49 years	Describe the participants included:		
	Are participants defined as women of reproductive age from 15–49 years?	Yes □ No □ Unclear □ Details:	
	How is the age or gender defined?	Details: Specific age group and gender (e.g. men / women):	
	*Do the participants meet the criteria for inclusion?*	Yes □ No □ →Exclude Unclear □	
Study setting: Developing countries Follow the list of all developing countries of Asia and Africa based on World bank definition of 2018	Is the study conducted in developing countries?	Yes □ No □ →Exclude Unclear □ Specify the region: –––––- Specify the country:–––––––-	
Intervention (Studies will be included that have measured the efficacy and effectiveness of iron)	Intervention		
	*Does the study measure the* efficacy and effectiveness of iron in reducing anemia* ?*	Yes □ No □ →Exclude Unclear □	
	*Does the study measure the* effects of iron (in any form) in reducing anemia* ?*	Yes □ No □ →Exclude Unclear□	
Types of outcome measures (anemia or hemoglobin levels): defined as Hb < 12.0 g/dl or Hct < 36% among non-pregnant women, and Hb < 11.0 g/dl or Hct < 33.0% among pregnant women	List outcomes:	Give definition of anemia used by author:	
	*Do the outcome measures meet the criteria for inclusion?*	Yes □ No □ →Exclude Unclear□	
Year of Publication 2000 to 2020	*Is the identified article published between 2000 and 2020?*	Yes □ No □ →Exclude Unclear □ Specify the year––––-	
Language of the published article English language	*Is the identified article published in English language?*	Yes □ No □ →ExcludeUnclear □ Specify the language––––	
Type of journal Peer reviewed journal (*Check from the list of all relevant journals or run a google search)*	*Is the identified journalpeer reviewed?*	Yes □ No □ →ExcludeUnclear □ Specify the *journal* ––––	
**Summary of Assessment for Inclusion**			
Include in review □	Exclude from review □		
Independently assessed by two authors, and then compared? Yes □ No □	Differences resolved Yes □ No □		
Notes:			

Eligibility criteria were categorized into four major headings using PICO (population, intervention, comparison, and outcome) algorithm (Table 2) [34]. The population for the current review was women of reproductive age (pregnant and non-pregnant women). The intervention was considered an iron supplement alone or in combination with folic acid or other micronutrients, taken orally or intravenously in a dosage of 60 to 100 mg daily for at least one month. To have the potential benefits of 60 to 100 mg of iron in reducing anemia and improving hemoglobin levels, WRA should have consumed iron for at least one month [35]. Since the evidence suggests that iron should be taken by WRA for at least 3 to 4 weeks to show its benefits, we decided to include RCTs where women were given iron for at least one month [35, 36]. Women in the control or comparison group received no iron supplement, a placebo, or any other mineral such as zinc, vitamin A, or B12 without iron or a different dose of iron than the defined intervention (details below). The primary outcome of the review is the improvement in the mean hemoglobin levels of women at the end of the intervention (details below). Lastly, the study design was either individual or cluster randomized controlled trials conducted in hospitals or communities. To include the most appropriate research articles that meet the eligibility criteria, we used filters on the year of publication (2000–2020), gender (females), age of study participants (15–49 years), language (English), and study designs (RCTs).

**Table 2:** Eligibility criteria according to the PICOS framework

Attribute	Inclusion Criteria	Exclusion Criteria
Population	All studies included women of reproductive age from 15 to 49 years of age Studies involving pregnant or non-pregnant women and married or non-married women of reproductive age from 15 to 49 years of age	Studies involving children or elderly under the age of 15 or over the age of 49 years Studies focused on men of any age
Intervention	All interventional (experimental) studies have measured the effect of iron therapy on the reduction of anemia	Studies have measured the impact of any other intervention (other than iron) on anemia
Comparison	The comparison group is the women who are given interventions other than iron or assigned to a placebo	Not applicable
Outcome	Anemia is measured objectively and defined as Hb < 12.0 g/dl or Hct < 36% among non-pregnant women, and Hb < 11.0 g/dl or Hct < 33.0% among pregnant women	Studies that have measured outcomes other than anemia such as nutritional deficiencies, food insecurity, etc. as a proxy indicator of anemia
Study Designs	Intervention Studies include both randomized and non-randomized controlled trials	Non-experiment observational quantitative studies (cross-sectional, case–control, cohort), pre-and post-test designs, commentaries, editorials, symposium proceedings, systematic reviews, secondary articles, and qualitative studies
Language	Studies available in the English Language	Studies that are not available in English translation
Period	Studies were published between January 2000 to December 2020 to capture a wide range of recently published literature	Studies published before January 2000
Type of journal	Studies published in peer-reviewed local and international journals	Studies published in non-peer-reviewed journals

### Intervention and primary outcome

Iron therapy in the form of tablets/ syrup/ injectables administered daily, weekly, or monthly in the case of injectables was proposed as an intervention. In addition, we considered interventions that evaluated the effect of iron and folic acid supplementation in addition to the usual iron-rich diet. The main outcome is a change in the mean hemoglobin level. We included RCTs that used hemoglobin levels (grams per deciliter (g/dL)) to label a WRA as anemic using the WHO proposed cut-offs of hemoglobin level of < 12.0 g/dL or Hct < 36% and < 11.0 g/dL or Hct < 33.0% for non-pregnant and pregnant women respectively (52). The secondary outcomes were the prevalence of anemia, mean serum ferritin, serum transferrin, and serum iron at the end of the intervention.

### Sources of information and search strategy

A systematic literature search was performed electronically to assess the efficacy of iron as a therapy to reduce anemia among WRA in LMICs. Two authors searched the databases independently for studies using a combination of search terms developed in response to the proposed research question. As a first step, four principal concepts, including (anemia, WRA, iron therapy, and LMICs) were identified. This was followed by using the synonyms of these major concepts, such as low hemoglobin/hematocrit level (anemia), married women/married pregnant women/ married non-pregnant women, iron/iron supplements/iron therapy/iron regimen (Intervention), and developing countries/poor-resource countries/ resource-constrained countries/less developed countries (LMICs). Besides, we used different spellings of these concepts, such as “anemia vs. anemia” and “hemoglobin vs. hemoglobin.” to obtain appropriate studies. Then, to find more research studies with the same root word, we combined the major concepts using combinations (AND, OR) and truncation (*). Further, indexed keywords in the Medical Subject Headings (MeSH) were used to ensure uniform search terms. Initially, the search strategy (shown in Table 3) was developed using the PICO framework. The search strategy was further refined, and finally, the below search strategy was used to search three databases.

“Iron therapy OR iron intervention OR oral iron [MeSH Terms]) AND (anemia* OR iron deficiency anemia*) AND (women OR married women* OR pregnant or non-pregnant women*) AND (poor countries OR developing countries OR low-middle-income countries)”.

**Table 3:** Search strategy according to PICO criteria

**Population**	‘women*’ [Mesh] OR ‘women*reproductive age*’ OR pregnant* OR married* OR non-pregnant* OR ‘married woman*’ OR ‘married pregnant woman’ OR ‘married non-pregnant woman’ OR ‘pregnant women’ ‘reproductive age’ OR ‘non-pregnant women’ ‘reproductive age’ [Mesh]) AND
**Intervention**	Iron supplements OR Iron therapy OR Iron tablets [MeSH Terms]) OR Iron fish [MeSH Terms]) OR Iron fortification [MeSH Terms]) OR iron medication [MeSH Terms]) OR iron in any form [MeSH Terms]) OR iron syrup [MeSH Terms]) OR iron rich diet[MeSH Terms]) OR iron rich fruits, vegetables, meat [MeSH Terms]) AND
**Comparison**	The comparison group is women who are given interventions other than iron or assigned to a placebo
**Outcome**	Anemia OR Hemoglobin levels OR Hemoglobin concentrations OR Hemoglobin status OR low Hemoglobin levels OR low Hemoglobin concentrations OR ‘low hematocrit levels’ OR Anemia symptoms OR paleness AND

### Study selection

**Fig. 1:**
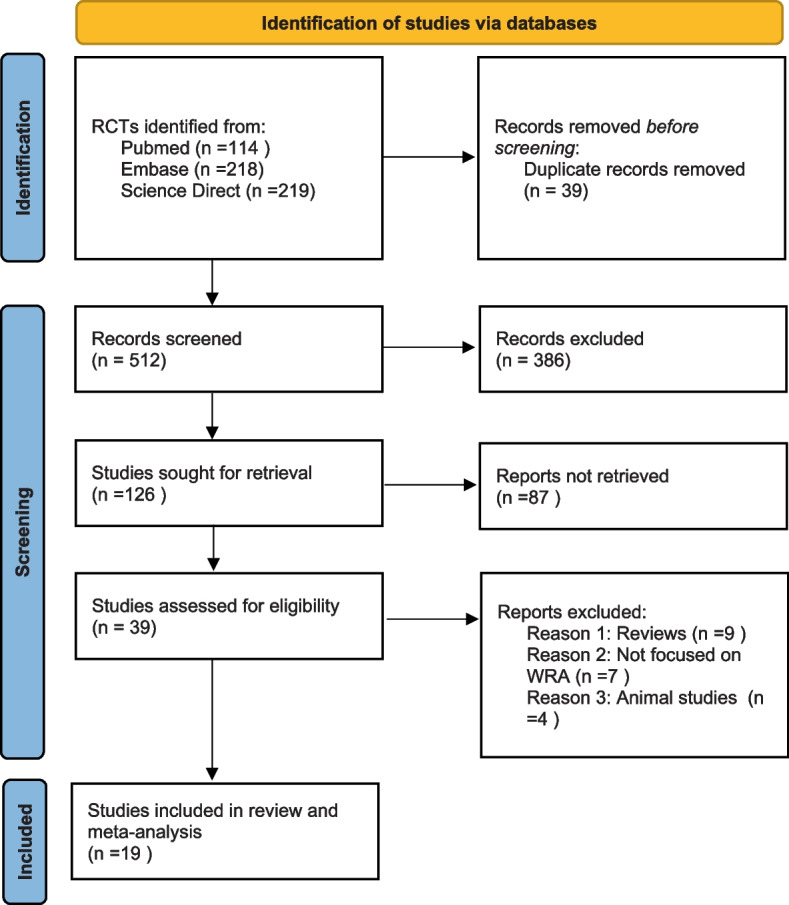
PRISMA 2020 flow diagram summarizing the identification and selection of relevant Randomized Controlled Trials

We used endnote software to handle research articles exported from the databases [37]. During the study selection process, title and abstract screening were conducted by two review authors independently. All research articles were screened by study title using Endnote software, followed by screening the study abstracts of the shortlisted articles. This was followed by full-text screening conducted by two review authors independently. The full texts of the shortlisted articles were then retrieved and screened against the inclusion and exclusion criteria. A third reviewer, an expert in the field, resolved disagreements between the two. Before ruling any study ineligible, both reviewers independently reviewed the full texts of the articles, and each reviewer provided strong justification. The third reviewer made the final decision to consider an article relevant. The flow diagram generated to illustrate the study selection process is shown in Fig. 1.

### Data collection process

Two independent reviewers completed a customized data extraction sheet for each eligible research article. To ensure that all important results and conclusions were considered in the review, the data extraction tables of the two independent reviewers were tallied. We enlisted the help of a third evaluator to resolve any conflicts or discordant information between the data extraction processes of two independent reviewers. Furthermore, existing research studies on the identified topic were reviewed to identify key items for the data extraction form. The data extraction form includes the following items: study name and author with publication year, study location, sample source, the sample size of both intervention and control groups, characteristics of study participants: baseline hemoglobin (g/dl), type of participants, age category, length of follow-up, intervention type and mode of administration, blinding procedure, randomization method, and key findings for the primary and secondary outcomes.

### Assessment of risk of bias

Overall quality was assessed using a revised Cochrane risk-of-bias tool for RCTs (RoB 2.0), which assesses “selection, performance, attrition, detection, and reporting bias by evaluating reported sequence generation, allocation concealment, blinding of participants and personnel, incomplete outcome data, selective outcome reporting, and other possible sources” [38]. Two independent review authors assessed the risk of bias. The conflict or disagreement between two independent review authors regarding the risk of bias assessment was resolved by a thorough discussion between two reviewers, and a third reviewer was invited if the two independent reviewers did not resolve the conflict. A high risk of bias was recognized if randomization or allocation concealment was either absent or judged at a higher risk, participants were not blinded, or there was high or imbalanced attrition across the groups. All RCTs that did not fulfill this criterion were categorized as studies with a low risk of bias. We synthesized the findings of the risk of bias, and the degree of bias was rated as low, high, or unclear. A final graph was generated to visualize the extent of bias in all eligible studies. Further, we assessed publication bias by constructing and assessing the asymmetry of funnel plots.

### GRADE assessment for overall certainty of the evidence

Using the GRADE (Grading of Recommendations, Assessment, Development, and Evaluation), we assessed the certainty of evidence [39]. The GRADE provides a reproducible and transparent framework to grade the certainty of evidence. Two independent review authors assessed GRADE. The GRADE assessment helped to rate the certainty of the evidence of iron therapy in improving anemia. The two authors independently assessed the certainty of the evidence for different outcomes such as serum hemoglobin, ferritin, iron, transferrin, and anemia. Since we included all RCTs in the review, the evidence was initially set as highly uncertain due to a lack of residual confounding. However, to increase our confidence for high certainty of the evidence, we used five additional criteria (risk of bias in individual RCTs), inconsistency of findings between RCTs, indirectness of evidence, imprecision of the estimate, and publication bias to make a conclusion about the overall certainty of evidence [39]. We ultimately developed a summary of findings (SoF) table using GRADE.

### A narrative synthesis of eligible studies

Before conducting a quantitative analysis of the same studies, we conducted a narrative synthesis of the eligible studies. To begin, we conducted a descriptive analysis on all of the final shortlisted full-text RCTs to collect information on the authors, publication year, study location, sample source, the sample size of both intervention and control groups, characteristics of study participants: baseline hemoglobin (g/dl), type of participants, age category, length of follow-up, intervention type and mode of administration, blinding procedure, and method of randomization. To begin, two reviewers thoroughly read the full text of the articles to extract data and summarize the key findings. The same reviewers then recorded analytical interpretations of the findings to identify important emerging themes. Finally, they highlighted the role of iron in decreasing anemia among WRA in LMICs by summarizing the relevant findings for the primary and secondary outcomes.

### Statistical analysis for quantitative results

Open Meta[analyst] software was employed to analyze the data retrieved from all eligible studies [40]. We used the standardized mean difference (SMD) and its respective 95% confidence interval (CI) to identify differences in hemoglobin mean across intervention and comparison groups. We used fixed effects (Mantel–Haenszel method) and random effects (DerSimonian and Laird method) meta-analysis models to compute summary estimates for the overall effect of iron. The primary analysis used a random effects model to generate a pooled effect estimate for the primary and secondary outcomes. Because of true differences in the prevalences of causal partners, it is reasonable to expect that effects will vary across populations. Since the eligible RCTs were from different international populations, using a random effects model was considered the most conservative analysis. Although we used a commonly used fixed effect model at first, the fixed effect model may understate the uncertainty of our findings [41]. Furthermore, the fixed effects model generated narrow confidence intervals that failed to account for actual variance between studies [41]. Also, the fixed model assumes that studies are drawn from populations with the same effect size, which may not be true for real-world data [42]. On the other hand, the random effect model assumes that studies are drawn from populations with varying effect sizes, which seems reasonable [42]. As a result, we kept the results from random-effects models rather than the fixed-effects model. Forest plots were created to visually inspect the degree of heterogeneity and demonstrate individual and pooled effects across eligible RCTs. Q-statistics and I^2^ were employed to quantify heterogeneity. Significant heterogeneity was found to exist when I^2^ was more than 50%, and a *p*-value of < 0.05 was used as a criterion for statistically significant results. Moreover, funnel plots were examined for asymmetry to identify publication bias. Because not all studies measured all iron deficiency markers, such as transferrin receptor or serum iron levels, we did not include these outcomes in the quantitative synthesis; instead, the findings were summarized using a narrative.

## Results

### Findings for the study selection process

Five hundred fifty-one RCTs were extracted from various databases, with 39 duplicates removed and 512 unique RCTs identified for further screening. Based on the eligibility criteria, 386 irrelevant titles and abstracts were removed after screening. After reviewing 126 identified abstracts, 87 studies were removed, including review articles, animal studies, secondary analysis, and in vitro studies. We read the full texts of 39 records and excluded 20 RCTs based on the inclusion and exclusion criteria. Finally, 19 studies were included in our review and meta-analysis (Fig. 1).

### Study characteristics of the eligible studies

A sample size of 19 studies ranged between 50 to 988 participants. However, the total sample size in the intervention (*n* = 2230) and control (*n* = 2281) groups was comparable. Most studies were conducted in India (*n* = 10), followed by Pakistan (*n* = 2) and Bangladesh (*n* = 2). One study was conducted in Nigeria (*n* = 1), Vietnam (*n* = 1), Tanzania (*n* = 1), Peru (*n* = 1), and Indonesia (*n* = 1). The majority of the studies were conducted in hospitals, including antenatal clinics and tertiary care OBGYN clinics (*n* = 14), followed by community-based sampling (*n* = 4) and participants from factory settings (*n* = 1). Baseline average hemoglobin (g/dl) for the intervention group ranged between 8.38 ± 1.41 to 12.5 ± 1.14, and for the control group, it ranged between 8.27 ± 1.20 to 11.8 ± 1.14. Most participants were aged 16–40 years, except for three studies, which had participants aged 15–49 years. Two studies included non-pregnant women, and nine included pregnant women ranging in gestation from 13–26 weeks (2nd trimester). Four studies included women with gestation periods ranging from 12–36 weeks, and two included pregnant women who were less than 24 weeks gestation at the time of enrollment. The intervention's effect was observed over a minimum of four weeks of gestation and a maximum of four weeks of the post-partum period in pregnant women (*n* = 17) and 13–26 weeks in non-pregnant women (*n* = 2) (Table 4).

**Table 4:** Characteristics of the included studies and their main findings for the primary and secondary outcomes (*n*=19)

**Study name**	**Age (years)**	**Study location**	**Baseline hemoglobin (mean ± SD)**		**Intervention group (n)**	**Comparison group (n)**	**Source of sample**	**Type of participants**	**Type of Intervention**	**Duration of intervention**	**Comparison group**	**Blinding procedure**	**Randomization method and level**	Primary outcome measure*	Secondary outcome measure**
			**Intervention group**	**Control group**											
Mumtaz et al. (2000) [43]	17 to 35 years	Peri-urban and rural Northern Pakistan	9.2 ± 1.4	9.5 ± 1.0	100	91	Tertiary care hospital	> 20 weeks of gestation	Daily iron supplementation capsules comprise 200 mg ferrous sulfate (60 mg elemental iron)	12 weeks of gestation	Twice-weekly iron supplementation capsules comprised of 200 mg ferrous sulfate (60 mg elemental iron)	Double-blind	Random number generation	Mean hemoglobin level was significantly higher in the intervention group (*p* < 0.001)	Serum Ferritin levels significantly increased in the intervention group
Zavaleta et al. (2000) [44]	15 to 35 years	Villa El Salvador/Lima/Peru	11.6 ± 1.2	11.5 ± 1.4	325	320	Hospital-based	10 to 24 weeks of gestation	Daily oral supplements of 60 mg Fe (ferrous sulfate) and 250 mg folic acid	10 to 24 weeks, 28 to 30 weeks 37to 38 weeks, 4 weeks postpartum	The same amount of iron and folic acid along with 15 mg Zn (as zinc sulfate)	Double-blind	Random assignment and stratification	Mean hemoglobin level was not significantly different in both the groups (*p* > 0.005)	No statistically significant difference was found in S. ferritin level and prevalence of anemia in both the groups
Ekström et al. (2002) [45]	Not mentioned	Rural areas/Mymensingh thana (subdistrict), Bangladesh	11.2 ± 1.3	11.0 ± 1.2	74	66	Antenatal center	18 to 24 weeks of gestation	Women received weekly 2 doses of supplements/tablets comprised of 60 mg Fe and 250 μg folic acid	12 weeks of gestation	Women received daily 1 dose of supplements/tablet comprised of 60 mg Fe and 250 μg folic acid	Not specified	Not specified	No significant difference was found in hemoglobin concentration between the two groups (*p* = 0.422)	No statistically significant difference was found in the prevalence of anemia between the two groups
Thuy et al. (2003) [46]	17 to 49 years	Vietnam	11.1 ± 0.8	11.0 ± 0.8	64	72	Factory setting	Non-pregnant women	Women received daily 10 mL of Iron-fortified fish sauce fortified with 10 mg Fe	6 months (26 weeks)	Women received daily 10 mL of non-fortified fish sauce	Double-blind	Not specified	Mean hemoglobin level was significantly higher in the intervention group (*p* < 0.0001)	S. Ferritin was statistically significantly higher in the intervention group. The prevalence of anemia was statistically significantly lower in the intervention group. S. transferrin receptor was statistically significantly lower in the intervention group
Makola et al. (2003) [47]	Not mentioned	Tanzania	10.5 ± 1.4	10.5 ± 1.5	127	132	Antenatal center	12 to 34 weeks of gestation	Micronutrient-fortified with 11 micronutrients including Fe	8 weeks of gestation	Non -fortified beverage (placebo)	Double-blind	Block randomization	Mean hemoglobin level was significantly increased in the intervention group (*p* = 0.015)	A statically significant increase in S. Ferritin was found in the intervention group
Mukhopadhyay et al. (2004) [48, 49]	Not mentioned	India	11.3 ± 1.4	11.6 ± 0.9	40	40	Antenatal clinic	< 20 weeks of gestation	Daily oral tablet of 100 mg elemental iron and 500 mg folic acid	32 to 34 weeks gestation	Weekly oral tablet of 200 mg elemental iron and 1000 mg folic acid	Single-blinded	Block randomization	Mean Hemoglobin level was not significantly different between two groups (*p* = 0.11)	No secondary outcome was assessed
Mukhopadhyay et al. (2004) [48, 49]	Not mentioned	New Delhi, India	11.6 ± 0.9	11.3 ± 1.0	40	40	Antenatal clinic	< 20 weeks of gestation	200 mg elemental iron tablets weekly	32 to 34 weeks of gestation	100 mg elemental iron tablets daily	Not specified	Block randomization	Mean hemoglobin level was not significantly differed in both the groups (*p* < 0.05)	Statistically significant decrease in S. Ferritin level in the intervention group and decrease in the prevalence of anemia in the control group
Sharma et al. (2004) [50]	18 to 40 years	New Delhi, India	9.4 ± 0.94	9.6 ± 0.87	100	100	Antenatal clinic	18 to 24 weeks of gestation	Three intramuscular doses of 250 mg elemental Fe as iron dextran at 1 month intervals plus oral doses of 5 mg folic acid twice weekly	37 to 41 weeks of gestation	Daily oral dose of 100 mg elemental Fe and 500 µg folic acid	Not specified	Partial randomization	Mean hemoglobin was improved in both the groups but the difference was insignificant (*p* > 0.005)	Statistically significant increase in S. Ferritin level in the intervention group. Statistical significant improvement in Serum iron in both the groups
Kumar et al. (2005) [51]	Not mentioned	India	9.89 ± 0.75	9.60 ± 0.77	75	75	Antenatal Clinic	16 to 24 weeks of gestation	Daily oral iron therapy of 100 mg of elemental iron	36 weeks of gestation	Overall, intramuscularly 2 doses of 250 mg of iron sorbitol with an interval of 4 to 6 weeks	Not specified	Not specified	Mean Hemoglobin was improved in the intervention group but the difference between the two groups was not statistically significant (*p* > 0.05)	S. Ferritin was statistically significantly increasing in control (parenteral iron) group. No significant difference was found in S. iron between the two groups
Saha et al. (2007) [52]	20 to 40 years	Chandigarh, India	8.47 ± 0.72	8.39 ± 0.74	48	52	Tertiary care hospital	14 to 27 weeks of gestation	One tablet once daily of Iron polymaltose Complex 100 mg elemental iron + folic acid 500 mcg for 8 weeks	27 weeks gestation	One tablet orally twice daily of Ferrous Sulphate 60 mg elemental iron + folic acid 500 mcg for 8 weeks	Double-blind	Not specified	Significant increase in the mean hemoglobin in both groups (*p* < 0.05)	Statistically significant increase in S. Ferritin level in both the groups
Bhutta et al. (2009) [53]	15 to 49 years	Urban and rural Sindh/Pakistan	10.7 ± 1.6	10.8 ± 1.5	466	522	Community-based	< 24 weeks of gestation	Multiple micronutrient supplements contained iron 30 mg (ferrous fumarate) and folic acid (400 μg), retinol (800 μg), zinc (15 mg), 2 mg of copper, 65 μg of selenium, and 150 μg of iodine, vitamins: D (200 IU), E (10 mg), C (70 mg), B1 (1.4 mg), B3 (18 mg), B2 (1.4 mg), B6 (1.9 mg), B12 (2.6 μg)	Post-natal visit	Iron (60 mg) and folic acid (400 μg) supplementation tablets	Double-blind	Block randomization	Mean hemoglobin level was not significantly different between the two groups (*p* = 0.27)	Statistically, significant improvement was found in S. Ferritin level in the intervention group
Wijaya-Erhardt et al. (2011) [54]	15 to 49 years	Karanganyar and Demak, of Central Java Province, Indonesia	12.5 ± 1.14	11.8 ± 1.14	110	117	Community-based	12 to 20 weeks of gestation	Optimized food was given 6 days per week comprised 600 g of tempeh, 30 g of meat, 350 g of guava, 300 g of papaya, and 100 g of orange along with tablets containing 60 mg of Fe and 250 mg of folic acid	36 weeks of gestation	Received tablets containing 60 mg of Fe and 250 mg of folic acid	Not specified	Cluster level	Mean hemoglobin level was decreased in both the groups (p < 0.05)	Statistically significant decrease in S. Ferritin level and S. Iron (Fe) level, a significant increase in transferrin receptor in both the groups
Choudhury et al. (2012) [55]	Not mentioned	Rural/ Central Bangladesh	10.9 ± 1.4	11.1 ± 1.3	207	198	Antenatal care centers	14 to 22 weeks of gestation	Micronutrient powder (contains 60 mg of elemental iron, 400 μg of folic acid, 30 mg of vitamin C, and 5 mg of zinc)	32 weeks of gestation	Iron and folic acid tablets (60 mg of elemental iron and 400 μg of folic acid)	Not specified	Cluster level	Mean hemoglobin was not significantly different in the intervention group (*p* = 0.106)	The prevalence of anemia improved in the intervention group but was statistically insignificant
Magon et al. (2014) [56]	18 to 35 years	Rajasthan/India	8.83 ± 1.7	8.38 ± 1.4	45	47	Community-based	14 to 16 weeks of gestation	Weekly distribution of leaf concentrate fortified ready-to-eat (lcRTE) snack in a dried powdered form fortified with 7 g Leaf concentrate	35–36 weeks of gestation	Weekly distribution of standard ready-to-eat (sRTE) snack contained 102 g wheat flour and 18 g soya flour	Single-blind	Consecutively numbered sealed envelopes along with block randomization	Mean hemoglobin level was improved in the intervention group significantly (*p* < 0.001)	-
Kamdi et al. (2015) [57]	18 to 30 years	Maharashtra and Gujarat, India	8.38 ± 1.41	8.27 ± 1.20	26	24	Health care facility based	12 to 26 weeks of gestation	A single daily dose of tablet ferrous asparto glycinate (FAG) (contains 100 mg of elemental iron + 300 μg of L-methyl folate + 500 μg of methylcobalamin)	28 days (4 weeks) of gestation	The single daily dose of tablet ferrous ascorbate (contains100 mg of elemental iron + 1.1 mg of folic acid)	Double-blind	Stratification and matching	Mean higher levels of hemoglobin in the intervention group (*p* < 0.01)	Statistically significant rise in S. Ferritin level in the intervention group
Mehta et al. (2017) [58]	18 to 35 years	India	10.5 ± 1.2	10.5 ± 1.3	65	71	Healthcare facility based	Non-pregnant women	One non-heme iron supplement bar (contain 14 mg Fe)/day (termed as GudNeSs bars)	90 days (13 weeks)	No intervention (No placebo either)	None	Cluster level	Mean hemoglobin level increased among intervention group (*p* < 0.001)	The prevalence of anemia became significantly lower in intervention group
	Not mentioned	India	9.38 ± 1	9.49 ± 1	184	184	Community based setting	12 to 16 weeks of gestation	Directly observed Iron Folic Acid (IFA) supplementation tablets once or twice daily	100 days(14 weeks) of gestation	Unobserved IFA supplementation tablets daily	Open-label	Block randomization	Mean hemoglobin level was higher in the intervention group (*p* < 0.001)	Serum ferritin and reduction of anemia in the intervention group was higher but not significant
	16 to 45 years	Nigeria	11.1 ± 0.9	11.0 ± 0.7	84	80	Antenatal clinic	14 to 24 weeks of gestation	Once daily FeSO4 200 mg supplements in tablets form (contains 65 mg elemental Fe)	37 weeks gestation	Twice daily FeSO4 200 mg supplements in tablets form (contain 130 mg of elemental iron)	Double blind	Balloting	Serum Hemoglobin was found to be lower among those on once daily dose as compared to twice daily (*p* = 0.002)	No difference in the serum ferritin levels between two groups
Jose et al. (2019) [59]	Not mentioned	India	8.57 ± 0.9	8.67 ± 0.8	50	50	Tertiary care hospital	16 to 36 weeks of gestation	Intravenous Ferric Carboxymaltose (FCM)	12 weeks gestation	Intravenous Iron sucrose complex (ISC)	Open-label	Computer generated block randomization	Mean rise in hemoglobin found in FCM group (*p* < 0.001)	No significant difference was found in S. Ferritin and S. Iron (Fe) level in both the groups

^*^Primary outcome measure was defined as change in Hemoglobin (Hb) level

^**^ Secondary outcome measures were defined as changes in the mean ferritin level, serum transferrin receptor, iron status, and iron deficiency

### Interventions included

Seven of the 19 RCTs were based on oral iron tablets to be administered daily [42–44, 48, 51, 52, 57, 60]. Two studies included oral iron tablets as a weekly dose [45, 49]. Two studies compared the efficacy of parenteral administration of iron supplementation [50, 59]. In one trial, ferric carboxymaltose was given intravenously (IV) and compared with IV iron sucrose complex [59]. Another trial provided an intervention based on three intramuscular doses of 250 mg elemental iron at intervals of 1 month along with the oral doses of 5 mg folic acid twice weekly and compared with a daily oral dose of 100 mg iron and 500 µg folic acid [50]. Of the total, three studies included micronutrient supplements comprised of zinc, minerals (copper, selenium), retinol, iodine, Vitamins: D, E, C, B1, B3, B2, B6, and B12 in different doses, along with iron and folic acid as an intervention and two out of three studies compared with iron (60 mg) and folic acid (400 μg) supplementation tablets and one study provided non-fortified beverage as a placebo [47, 53, 55]. Four studies out of 19 included an optimized preparation based on food and snacks in the form of bars, sauce, fortified ready-to-eat snacks in dried powdered form, and one study included meat, fresh fruits, and vegetable along with iron tablets (60 mg), and folic acid (250 mg) as shown in Table 4 [46, 54, 56, 58].

### Pooled effect for outcomes measurement

#### Pooled effect for the primary outcome, serum hemoglobin

**Fig. 2:**
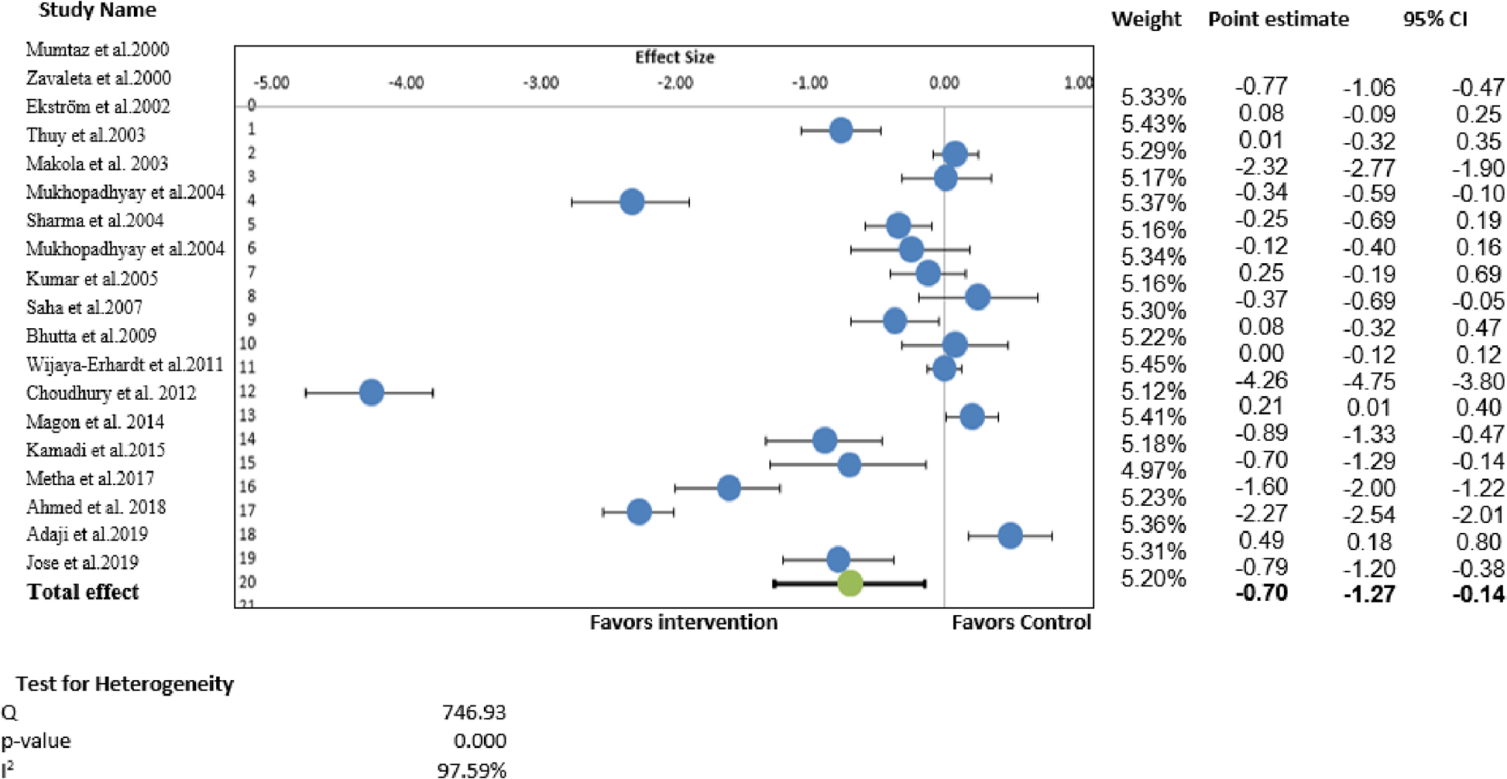
Forest plot summarizing the overall pooled effect of iron supplementation on the primary outcome, hemoglobin (*n* = 19)

Since the complete data were only available for the hemoglobin, we performed quantitative analysis for the primary outcome (hemoglobin) and one secondary outcome (Serum ferritin). A total of 19 studies (*n* = 4421 participants) were included in the meta-analysis to estimate the effect size. Meta-analysis indicated overall pooled effect estimate for the role of iron therapy in lowering the burden of anemia among the WRA group was -0.71 (95% CI: -1.27 to -0.14) (*p* = 0.008). The heterogeneity (I2) across included studies was found to be statistically significant, as indicated by the parameters of heterogeneity (Q = 746.93, I2 = 97.59%, *p* = 0.000) (Fig. 2).

Figure 2 depicts the overall findings showing that iron therapy positively affects hemoglobin levels. More specifically, iron therapy in any form increased mean hemoglobin levels by 0.40 gms. The intervention arm's mean and standard deviation for hemoglobin was 11.14 ± 1.11 gm/dl, while the comparison group's mean and standard deviation for hemoglobin was 10.74 ± 1.019 gm/dl. However, there were variations in the results of individual studies. For instance, nine studies found that mean hemoglobin levels improved in the intervention group significantly [43, 46, 47, 52, 56–60]. Eight RCTs found that the mean hemoglobin level was not significantly different between the two groups post-intervention [44, 45, 48–51, 53, 55]. One study showed that the mean hemoglobin level decreased significantly in both groups [54]. Another study documented that the hemoglobin level was decreased in the intervention group after comparing once-daily (intervention group) vs. twice-daily (control group) oral supplementation [42].

### Findings for the secondary outcomes

The included studies assessed secondary outcomes, including changes in mean serum ferritin level, serum transferrin receptor, serum iron, and improvement in iron deficiency anemia.

#### Pooled effect for the secondary outcome, serum ferritin levels

**Fig. 3:**
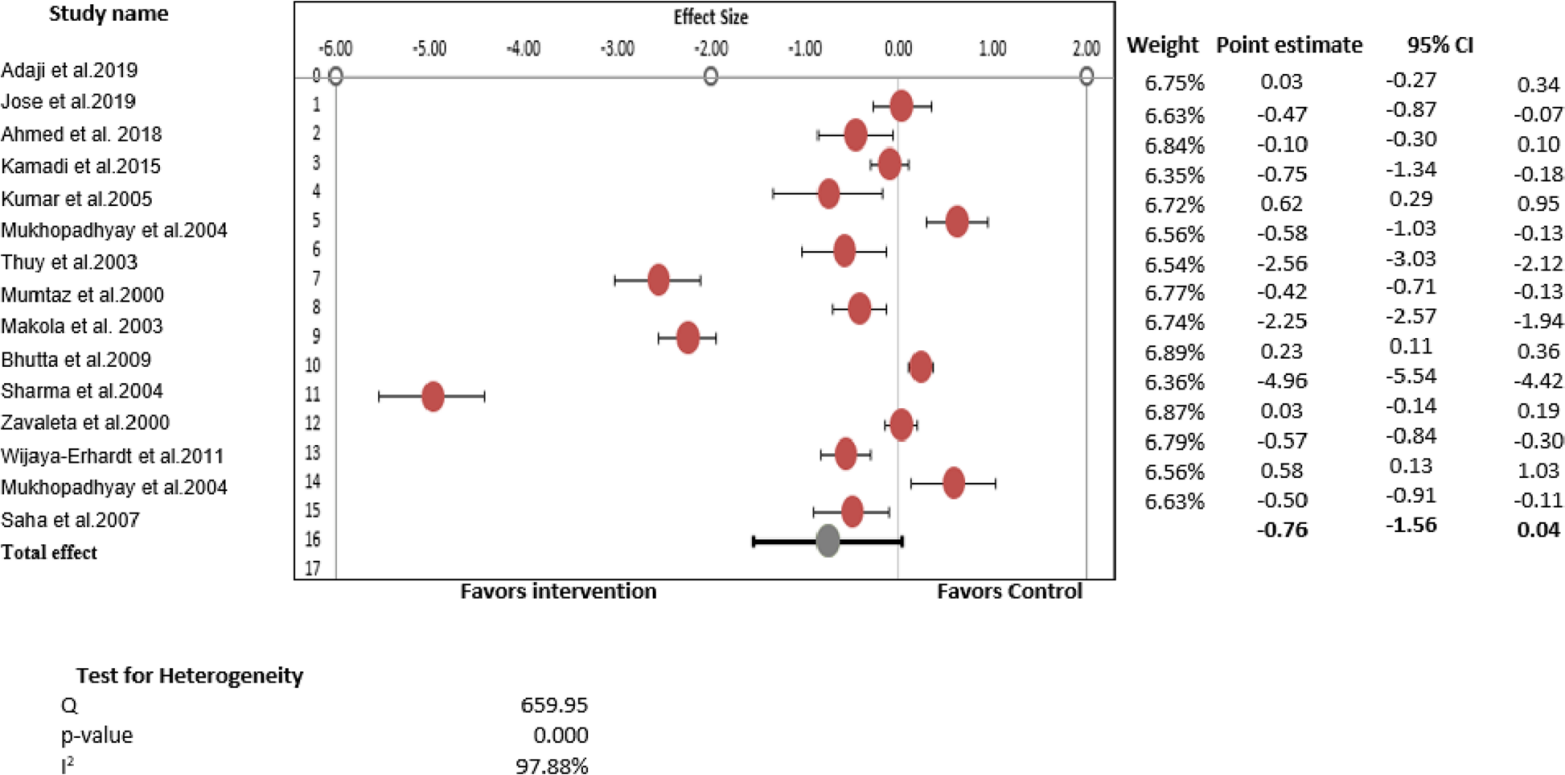
Forest plot summarizing the overall pooled effect of iron supplementation on the secondary outcome, Serum Ferritin (*n* = 15)

For the secondary outcomes, complete data were only available for serum ferritin; therefore, we performed a quantitative analysis for serum ferritin. A total of 15 studies (*n* = 3648 participants) were included in the meta-analysis to estimate the effect size. Meta-analysis indicated overall pooled effect estimate for the role of iron therapy in improving the ferritin levels among WRA was -0.76 (95% CI: -1.56 to 0.04) (*p* = 0.04). The heterogeneity (I^2^) across included studies was found to be statistically significant, as indicated by the parameters of heterogeneity (Q = 659.95, I^2^ = 97.88%, *p* = 0.000) (Fig. 3).

Overall, the results revealed a favorable effect of iron therapy in improving serum ferritin levels, as shown in Fig. 3. More precisely, in simple terms, iron therapy in any form improved the mean ferritin levels by 5.90ug/L. The mean and standard deviation for ferritin levels of the intervention arm was 37.61 ± 20.0, whereas the mean and standard deviation for ferritin levels of the comparison group was 31.71 ± 20.13. However, there were variations in the results of individual studies.

#### Change in mean serum ferritin levels: qualitative synthesis

Fourteen studies measured mean serum ferritin levels after the intervention as a secondary outcome. Six of the fourteen studies found statistically significant improvements in mean serum ferritin levels in the intervention group versus the control group [43, 46, 47, 50, 53, 57]. One RCT found significant improvement in the mean serum ferritin levels across both groups [52]. In contrast, one study documented a significant increase in the mean serum ferritin levels in the control group [51]. One RCT found a significant reduction in the mean serum ferritin levels in both groups [54], whereas one RCT noticed a substantial reduction in the mean serum levels in the intervention group than the control group [49]. 4 RCTs identified no significant difference in the mean serum ferritin levels between the two groups [42, 44, 59, 60].

#### Reduction in the anemia prevalence

Six randomized controlled trials investigated the reduction in the prevalence of anemia as an outcome of interest. Two out of six RCTs reported a significant decrease in the prevalence of anemia in the intervention group compared to the control group [46, 58]. While two other RCTs showed an improvement in anemia in the intervention than the control group, the findings were statistically non-significant [55, 60]. Two RCTs did not find a significant difference in the prevalence of anemia across both groups [44, 45]. And one RCT showed negative findings, meaning a significant decline in the prevalence of anemia was observed in the control group than in the intervention group, as shown in Table 4 [51].

#### Change in mean serum iron

Four out of 19 RCTs assessed change in serum iron levels post-intervention, and two trials reported no significant difference in the mean serum iron between both groups [51, 59]. One RCT reported significant improvement in the mean serum iron levels in both groups [50]. In contrast, another RCT identified a statistically significant reduction in the mean serum iron in both groups [54].

#### Change in serum transferrin receptor

Two of the 19 RCTs examined the change in serum transferrin receptor after an intervention, with one finding that serum transferrin receptor was significantly lower in the intervention group than in the control group [46]. However, another RCT reported increased serum transferrin receptors in both groups [54].

### Overall Quality assessment for RCTs

Table 5 depicts the overall quality assessment of the eligible RCTs. Overall, 6 of 19 studies were deemed to have a low risk of bias based on criteria such as the low risk of randomization, allocation concealment, or blinding [42–44, 53, 54, 57]. Of the total, seven studies were found to have a high risk of bias since the randomization method was unclear [45, 46, 51, 52, 54, 55, 58]. A high risk of bias was found in 2 studies where no allocation concealment was done [58, 59], and in 8 studies, concealment was unclear [45–51, 55]. The absence of blinding was found in 4 studies [56, 58–60]. An unclear description of blinding study participants or outcome assessors was found in 5 studies [45, 48–51, 54, 55] and, subsequently, labeled as having a high overall risk of bias.

**Table 5:** Risk of bias assessment of the studies included in the meta-analysis (*n* = 19)

	**Randomization Method**	**Allocation Concealment**	**Blinding of participants and personnel**	**Blinding of outcome assessors**	**Incomplete outcome data**	**Selective outcome reporting**	**Other bias**
Mumtaz et al. 2000 [43]							
Zavaleta et al. 2000 [44]							
Ekström et al. 2002 [45]							
Thuy et al. 2003 [46]							
Makola et al. 2003 [47]							
Mukhopadhyay et al. 2004 [48, 49]							
Mukhopadhyay et al. 2004 [48, 49]							
Sharma et al. 2004 [50]							
Kumar et al. 2005 [51]							
Saha et al. 2007 [52]							
Bhutta et al. 2009 [53]							
Wijaya-Erhardt [54]							
Choudhury et al. 2012 [55]							
Magon et al. 2014 [56]							
Kamdi et al. 2015 [57]							
Mehta et al. 2017 [58]							
							
							
Jose et al. 2019 [59]							

### Publication bias

**Fig. 4:**
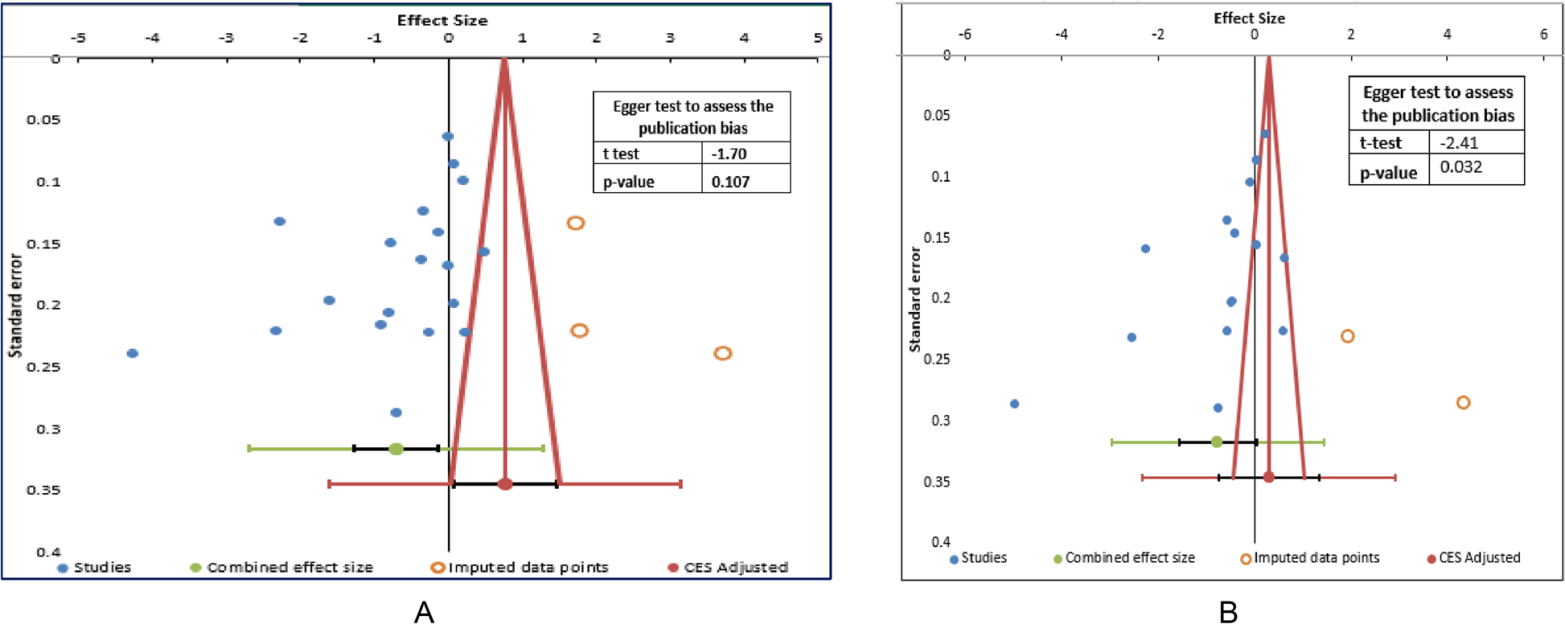
**A** Funnel plot to evaluate the publication bias among included studies in the meta-analysis for the primary outcome, hemoglobin (*n* = 19). **B** Funnel plot to evaluate the publication bias among included studies in the meta-analysis for the secondary outcome, Serum Ferritin (*n* = 15)

A graphical representation, a funnel plot, was employed to assess publication bias, indicating potential bias because the shape of the graph is not symmetrical, as shown in Fig. 4a and b for studies that assessed primary (hemoglobin) and secondary outcomes (ferritin), respectively. Since the funnel plot is subjective, we used a confirmatory objective statistical test (i.e., Egger’s test) to evaluate the publication bias. The objective test results for the primary outcome revealed no publication bias as the t-test was not statistically significant (Egger t-test = -1.7, *p* = 0.107), as shown in Fig. 4a. However, the same objective test was statistically significant for the serum ferritin (Egger t-test = -2.41, *p* = 0.032), suggesting a publication bias (Fig. 4b).

### Summary of findings using GRADE assessment

Since the meta-analysis was conducted on RCTs, the overall certainty of the evidence was considered reasonable. However, using the additional five criteria of GRADE, the certainty of the evidence was assessed for five outcomes, and SoF is provided in Table 6. The findings reveal overall certainty of the evidence for outcomes such as serum hemoglobin, serum ferritin, and anemia prevalence was moderate. This implies that the two independent authors believe that the truth or true effect size is probably close to the estimated effect size, and future research for similar outcomes may change the estimate for the effect size. However, the certainty of the evidence for outcomes such as serum iron and transferrin receptor was low, suggesting that further research for these two outcomes will likely change the estimate of the effect.

**Table 6:** Summary of findings (SoF) table illustrating the summary on certainty of overall evidence for five outcomes using GRADE

Outcomes	Number of participants (RCTs)	Effect size and 95% CI	Certainty of the evidence (GRADE)^a^	Comments
Mean Serum hemoglobin	4421 (19)	-0.71 (-1.27 to -0.14)	Moderat	The assessment of certainty by two authors concludes that effect size for the serum hemoglobin is precise and consistent with low risk of bias at outcome level, suggesting that the true effect size for serum hemoglobin is probably close to the estimated effect
Mean Serum Ferritin	3648 (15)	-0.76 (-1.56 to 0.04)	Moderate	The authors believe that effect size for the serum ferritin is precise and consistent with low risk of bias at outcome level, suggesting that the true effect size for serum ferritin is probably close to the estimated effect
Anemia Prevalence	1910 (6)	Could not be estimated	Moderate	While the effect size for anemia could not be calculated, the authors believe that narrative regarding anemia prevalence suggested that anemia declined in the intervention group. Since the evidence was direct, consistent with no risk of bias at the outcome level, the authors believe that certainty of evidence is moderate for the anemia outcome
Mean serum iron levels	677 (4)	Could not be estimated	Low	Since the authors could not estimate the effect size and the findings for iron are from very few studies with inconsistent results, the authors believe that certainty of evidence for iron is low and needs to be explored more in the future
Mean serum transferrin receptor levels	363 (2)	Could not be estimated	Low	Since the authors could not estimate the effect size for the serum transferrin and the findings for serum transferrin are from very few studies with inconsistent results, the authors believe that certainty of evidence for serum transferrin is low and needs to be explored more in the future

Very low: The truth or true effect size is totally different from the estimated effect size

Low: The true effect may be markedly different from the estimated effect size

Moderate: The authors believe that the truth or true effect size is probably close to the estimated effect size

High: The authors’ confidence is high, and they believe that the truth or true effect size is very similar to the estimated effect size

^a^GRADE certainty ratings

## Discussion

We conducted this systematic review and meta-analysis to assess the role of iron therapy in reducing anemia in pregnant and non-pregnant women. The review's findings showed that iron supplementation helps to improve markers of iron deficiency anemia, such as serum ferritin and hemoglobin levels. The current systematic review and meta-analysis findings are consistent with previous reviews. Low Yuan et al., for example, conducted a review in 2016 to assess the effectiveness of iron therapy in reducing anemia in menstruating women [29]. The authors demonstrated that daily iron supplementation reduces the burden of anemia and iron deficiency, raises hemoglobin levels, increases iron stores, improves exercise ability, and decreases fatigue. [29]. Likewise, another review conducted five years ago also revealed similar findings, where authors found positive effects of iron on hematological and pregnancy outcomes [30]. However, this review relied on studies with very low-quality evidence [30]. A review by Haider et al., carried out in 2013, showed consistent findings. Haider et al. emphasized the role of iron in improving birth weight rather than intermediate outcomes such as improving anemia or markers of iron deficiency [31]. Regardless of these differences, the analogous findings across these reviews indicate the role of iron in improving hemoglobin and other markers of iron-deficiency anemia among WRA.

The current review and meta-analysis findings are biologically plausible, and several mechanisms regulating iron absorption have been explored in different studies [61–63]. More precisely, the findings suggest that daily iron supplementation appears to be an effective intervention to reduce the burden of anemia among WRA. This review's findings complement those of other studies examining the role of iron supplements in reducing anemia in pregnant and non-pregnant women. Based on the results of this review and reasonably comparable findings from other reviews, iron, in any form, for pregnant or non-pregnant women may be beneficial in reducing the burden of maternal anemia. The question arises of how such intervention improves hemoglobin levels. Iron absorption in intestinal cells, followed by iron transfer to bone marrow, muscles, and other tissues, could be one possible answer to the proposed question. Iron is taken up by receptors in these tissues and used for various biological functions or stored [64]. Both animal and human studies reveal an inverse relationship between iron status or stores and the ability to absorb iron from intestinal cells [65]. This implies that an iron-deficient woman has the potential to absorb iron two times more than an iron-non-deficient woman because iron deficiency induces changes in the transport of iron across the intestine [65].

Furthermore, the evidence suggests that an iron-deficient individual should aim to increase hemoglobin concentrations by 1 g/dl every week and be aware of the dietary sources that inhibit iron absorption [29]. Iron absorption from supplements follows the same principles as iron absorption from dietary sources, and consuming vitamin C, meat, and acidic foods increases iron absorption [66]. Tannins, calcium, and phytates reduce the absorption and should not be consumed alongside iron [66]. In addition to the dietary resources, one needs to be aware of the geographical regions before providing iron. For example, health professionals may need to treat malaria in areas with endemic malaria while providing iron therapy to women [67]. Similarly, iron may also be affected by worm infestation. Therefore, healthcare professionals should consider deworming women before prescribing iron therapy to WRA [68]. This suggests that iron therapy may be beneficial when healthcare professionals become cognizant of the facts mentioned above while prescribing iron therapy to a woman.

Evidence from epidemiological studies shows that women from LMICs enter pregnancy with limited iron stores and lower hemoglobin levels than those from high-income countries [69]. Therefore, the demand for iron absorption is higher in women from resource-poor settings with a greater hematological response [70]. In addition, improving iron stores during pregnancy may also decrease the risk of mortality resulting from hemorrhage and may lead to improved hemoglobin and iron levels after pregnancy [9].

### Strengths and limitations

This review's main strength is that it provides insights into the effect of iron on a wide range of outcomes, including hemoglobin, serum ferritin, iron, transferrin receptor, and anemia prevalence. In addition, unlike other reviews, we included all studies from LMICs that looked at the role of iron therapy in any form among pregnant and non-pregnant women. In addition, by including only RCTs, the problem of the unknown and unmeasured confounding could be addressed, thereby improving the confidence in the validity of the findings. Additionally, no significant publication bias was found in our meta-analysis, indicating that most trials with positive, negative, or null findings were published in the literature.

However, some inherent limitations of the individual eligible studies need to be considered while interpreting the findings of this review. For example, high-quality studies included in this review and meta-analysis were relatively less (*n* = 5) because of methodological issues in the randomization methods, no or unclear allocation concealment, and lack of blinding. Moreover, the heterogeneity was found to be very high, which could be explained by factors such as variation in sample size, differences in the follow-up time, differences in the populations (pregnant and non-pregnant women), and substantial variations in the interventions designed (difference in dosages and composition, and duration of interventions). There was a significant variation in the given interventions, for example, daily iron supplementation vs. weekly, once-daily vs. twice daily, oral iron vs. parenteral iron, micronutrient powder and/or iron vs. only iron, fortified snacks in addition to iron vs. placebo, resulting in heterogeneous exposure. In addition, we included studies from 2000 to 2020, which is a relatively long period with a greater degree of variation in the methods of different RCTs. The purpose of including the more extended period was to capture multiple RCTs on the role of iron in improving anemia among WRA. However, considering a longer period may lead to a more significant heterogeneity due to a wide variation in the methods. While it may be challenging to avoid heterogeneity entirely, RCTs can be designed efficiently to assess the role of uniform dose and form of iron and to follow women for the same time in different settings. This will aid in determining the effect of only iron supplements versus a placebo in the control group to isolate the effect of a fixed dose of iron in reducing anemia. Furthermore, although we identified potential secondary outcomes such as iron and transferrin receptors, most RCTs did not measure the secondary outcomes we chose. Hence, the effect size for outcomes sucn as iron and transferrin receptors could not be estimated. Finally, we only included RCTs published in English, limiting our ability to have inferences from studies published in other languages.

## Conclusions

This review aimed to determine the effect of iron on hemoglobin levels and anemia in women of reproductive age. Overall, the review found that iron therapy, in any form, increases hemoglobin levels in pregnant and non-pregnant women and reduces iron deficiency anemia, as evidenced by increases in hemoglobin, serum ferritin, and decreased soluble transferrin receptors. The review also revealed that WRA in resource-constrained settings could be given iron in any form. The findings of this systematic review and meta-analysis may help physicians, researchers, and policymakers make informed decisions about providing iron therapy to pregnant and non-pregnant women and prepare them with enough iron stores for adequate fetal growth.

### Clinical and research implications

The current review and meta-analysis findings can be used to treat anemia among WRA in LMICs using simple iron therapy in oral or injectable forms, depending on the severity of anemia and a woman's needs. Simple, cost-effective, and culturally appropriate iron and folic acid therapies can be given to women before or during pregnancy to reduce the anemia burden. This improves hemoglobin and iron stores, as evidenced by an increase in ferritin levels, a marker of iron stores.

Although current evidence suggests that iron is important for WRA in LMICs, more research is needed to fill important gaps. For example, none of the eligible RCTs examined the underlying mechanisms by which iron can improve outcomes. There is a need to evaluate the benefits and side effects of iron and adherence to iron intake because there is a dearth of evidence on these outcomes. Further, almost all of the RCTs focused on improving iron status during pregnancy rather than during a critical preconception window when a woman can lay a good foundation for an upcoming baby by eating iron-rich foods or taking iron supplements. Thus, more research is needed in public health settings to assess the role of iron before and during pregnancy on iron markers as well as distal birth outcomes such as birth length and birth weight.

In LMICs, iron supplementation is usually combined with folic acid and/or other micronutrients. As a result, it is unclear how much improvement in anemia can be attributed to iron alone. Thus, more well-designed RCTs are needed to fully understand the efficacy and safety of iron alone in reducing anemia among WRA in LMICs. Finally, secondary outcomes such as serum ferritin, serum transferrin receptor, and transferrin saturation indicate a long-standing and sustained parameter of storage iron, and their depletion can result in iron deficiency. Hence, RCTs should measure these outcomes rigorously after interventions are given for a reasonable time to identify long-term iron stores [71]. Furthermore, serum ferritin, serum transferrin receptor, and transferrin saturation have been shown to be more accurate indicators of iron deficiency anemia. As a result, estimating these parameters will reveal long-term benefits [72].

## Supplementary Information

**Additional file 1. **Data extracted for Risk of Bias Assesment.

## Data Availability

This review was based on the synthesis of findings from the existing published RCTs and the references of those RCTs are mentioned in the reference list. Anyone who needs raw data of individual studies can directly contact the authors of individual studies or can retrieve the data from the published articles. All RCTs are properly cited in the references and a reference list can be used to access the RCTs online. However, we have uploaded the supplementary files of extracted data that were used for the analysis and risk of bias assessment.

## Abbreviations

LMICs: Low-middle-income countries
LBW: Low Birth Weight
PICOS: Population, Intervention, Comparison, Outcome, and Setting
PRISMA-P: Preferred Reporting Items for Systematic Reviews and Meta-Analyses Protocols
RCTs: Randomized Controlled Trials
WHO: World Health Organization
WRA: Women of Reproductive Age

## References

[1] Milman N (2011). Anemia—still a major health problem in many parts of the world!. Ann Hematol.

[2] Khan AM, Kidwai SS, Akhtar S, Ara J (2018). Knowledge, apptitude and practice: smoking and gutka habits in a lower socio-economic cohort. Int J Res Med Sci.

[3] Beckert RH, Baer RJ, Anderson JG, Jelliffe-Pawlowski LL, Rogers EE (2019). Maternal anemia and pregnancy outcomes: a population-based study. J Perinatol..

[4] Hare GM, Freedman J, Mazer CD (2013). risks of anemia and related management strategies: can perioperative blood management improve patient safety?. Can J Anesthesia/J Can d'anesthésie.

[5] Kavle JA, Stoltzfus RJ, Witter F, Tielsch JM, Khalfan SS, Caulfield LE (2008). Association between anaemia during pregnancy and blood loss at and after delivery among women with vaginal births in Pemba Island, Zanzibar, Tanzania. J Health Popul Nutr.

[6] Tunkyi K, Moodley J (2018). Anemia and pregnancy outcomes: a longitudinal study. J Matern Fetal Neonatal Med.

[7] Black RE, Victora CG, Walker SP, Bhutta ZA, Christian P, De Onis M, Ezzati M, Grantham-McGregor S, Katz J, Martorell R (2013). Maternal and child undernutrition and overweight in low-income and middle-income countries. Lancet.

[8] Rahman MM, Abe SK, Rahman MS, Kanda M, Narita S, Bilano V, Ota E, Gilmour S, Shibuya K (2016). Maternal anemia and risk of adverse birth and health outcomes in low-and middle-income countries: systematic review and meta-analysis, 2. Am J Clin Nutr.

[9] Xiong X, Buekens P, Alexander S, Demianczuk N, Wollast E (2000). Anemia during pregnancy and birth outcome: a meta-analysis. Am J Perinatol.

[10] Daru J, Zamora J, Fernández-Félix BM, Vogel J, Oladapo OT, Morisaki N, Tunçalp Ö, Torloni MR, Mittal S, Jayaratne K (2018). Risk of maternal mortality in women with severe anaemia during pregnancy and post partum: a multilevel analysis. Lancet Glob Health.

[11] Organization WH (2017). The global prevalence of anaemia in 2011.

[12] Sifakis S, Pharmakides G (2000). Anemia in pregnancy. Ann N Y Acad Sci.

[13] Ouédraogo S, Koura GK, Bodeau-Livinec F, Accrombessi MM, Massougbodji A (2013). Cot MJTAjotm, hygiene: Maternal anemia in pregnancy: assessing the effect of routine preventive measures in a malaria-endemic area. Am J Trop Med Hyg.

[14] Pasricha SR, Drakesmith H, Black J, Hipgrave D, Biggs BA (2013). Control of iron deficiency anemia in low- and middle-income countries. Blood.

[15] Organization WH (2015). The global prevalence of anaemia in 2011. The global prevalence of anaemia in 2011. edn.

[16] Unicef U, WHO U (2001). Iron deficiency anaemia: assessment, prevention, and control. A guide for programme managers.

[17] Organization WH (2014). WHA Global Nutrition Target 2025: Anemia Policy Brief. Global Nutr Target.

[18] Bentley M, Griffiths P (2003). The burden of anemia among women in India. Eur J Clin Nutr.

[19] Panja TK, Sinha NK, Chakrabortty S, Maiti S, Dutta D, Kundu P, Pal S (2019). Prevalence of anaemia in varied nutritional state among the women of reproductive ages belonging to low socioeconomic status of rural India. Age (year)..

[20] Panyang R, Teli AB, Saikia SP (2018). Prevalence of anemia among the women of childbearing age belonging to the tea garden community of Assam, India: A community-based study. J Family Med Primary Care.

[21] Addis Alene K, Mohamed Dohe A. Prevalence of anemia and associated factors among pregnant women in an urban area of Eastern Ethiopia. Anemia. 2014;2014. 10.1155/2014/561567 PMC4158560 25215230

[22] Getachew M, Yewhalaw D, Tafess K, Getachew Y, Zeynudin A (2012). Anaemia and associated risk factors among pregnant women in Gilgel Gibe dam area, Southwest Ethiopia. Parasit Vectors.

[23] Haidar J (2010). Prevalence of anaemia, deficiencies of iron and folic acid and their determinants in Ethiopian women. J Health Popul Nutr.

[24] Chowdhury HA, Ahmed KR, Jebunessa F, Akter J, Hossain S, Shahjahan M (2015). Factors associated with maternal anaemia among pregnant women in Dhaka city. BMC Womens Health.

[25] Camaschella C (2015). Iron-deficiency anemia. N Engl J Med.

[26] Lopez A, Cacoub P, Macdougall IC, Peyrin-Biroulet L (2016). Iron deficiency anaemia. Lancet.

[27] World Health Organization. Guideline: intermittent iron and folic acid supplementation in menstruating women. World Health Organization; 2011. 24479204

[28] Organization WH (2014). Global nutrition targets 2025: Breastfeeding policy brief.

[29] Low MS, Speedy J, Styles CE, De-Regil LM, Pasricha SR (2016). Daily iron supplementation for improving anaemia, iron status and health in menstruating women. Cochrane Database Syst Rev.

[30] Peña-Rosas JP, De-Regil LM, Garcia-Casal MN, Dowswell T (2015). Daily oral iron supplementation during pregnancy. Cochrane Database Syst Rev.

[31] Haider BA, Olofin I, Wang M, Spiegelman D, Ezzati M, Fawzi WW (2013). Anaemia, prenatal iron use, and risk of adverse pregnancy outcomes: systematic review and meta-analysis. BMJ.

[32] Pasricha SR, Low M, Thompson J, Farrell A, De-Regil LM (2014). Iron supplementation benefits physical performance in women of reproductive age: a systematic review and meta-analysis. J Nutr.

[33] Page MJ, McKenzie JE, Bossuyt PM, Boutron I, Hoffmann TC, Mulrow CD, Shamseer L, Tetzlaff JM, Akl EA, Brennan SE (2021). The PRISMA 2020 statement: an updated guideline for reporting systematic reviews. BMJ (Clin Res Ed).

[34] Methley AM, Campbell S, Chew-Graham C, McNally R, Cheraghi-Sohi S (2014). PICO, PICOS and SPIDER: a comparison study of specificity and sensitivity in three search tools for qualitative systematic reviews. BMC Health Serv Res.

[35] Knudsen VK, Hansen HS, Ovesen L, Mikkelsen TB, Olsen SF (2007). Iron supplement use among Danish pregnant women. Public Health Nutr.

[36] Boti N, Bekele T, Godana W, Getahun E, Gebremeskel F, Tsegaye B, Oumer B. Adherence to Iron-Folate supplementation and associated factors among Pastoralist’s pregnant women in Burji districts, Segen area People’s zone, southern Ethiopia: community-based cross-sectional study. Int J Reprod Med. 2018;2018. 10.1155/2018/2365362 PMC6333009 30693285

[37] Yali Z (2004). The Identification and Evaluation of the Kernel Authors of New Technology of Library and Information Service. Data Anal Knowledge Discov.

[38] Higgins J, Wells G (2011). Cochrane handbook for systematic reviews of interventions.

[39] Guyatt GH, Oxman AD, Vist GE, Kunz R, Falck-Ytter Y, Alonso-Coello P, Schünemann HJ (2008). GRADE: an emerging consensus on rating quality of evidence and strength of recommendations. BMJ.

[40] Wallace BC, Schmid CH, Lau J, Trikalinos TA (2009). Meta-Analyst: software for meta-analysis of binary, continuous and diagnostic data. BMC Med Res Methodol.

[41] Overton RC (1998). A comparison of fixed-effects and mixed (random-effects) models for meta-analysis tests of moderator variable effects. Psychol Methods.

[42] AdajiJAJFoO (2016). Gynaecology: Daily Versus Twice Daily Dose of Ferrous Sulphate Supplementation in Pregnant Women: A Randomised Clinical Trial.

[43] Mumtaz Z, Shahab S, Butt N, Rab MA, DeMuynck A (2000). Daily iron supplementation is more effective than twice weekly iron supplementation in pregnant women in Pakistan in a randomized double-blind clinical trial. J Nutr..

[44] Zavaleta N, Caulfield LE, Garcia T (2000). Changes in iron status during pregnancy in Peruvian women receiving prenatal iron and folic acid supplements with or without zinc. Am J Clin Nutr..

[45] Ekström E-C, Hyder SZ, Chowdhury AMR, Chowdhury SA, Lönnerdal B, Habicht J-P, Persson LÅ (2002). Efficacy and trial effectiveness of weekly and daily iron supplementation among pregnant women in rural Bangladesh: disentangling the issues. Am J Clin Nutr..

[46] Thuy PV, Berger J, Davidsson L, Khan NC, Lam NT, Cook JD, Hurrell RF, Khoi HH (2003). Regular consumption of NaFeEDTA-fortified fish sauce improves iron status and reduces the prevalence of anemia in anemic Vietnamese women. Am J Clin Nutr..

[47] Makola D, Ash DM, Tatala SR, Latham MC, Ndossi G, Mehansho H (2003). A micronutrient-fortified beverage prevents iron deficiency, reduces anemia and improves the hemoglobin concentration of pregnant Tanzanian women. J Nutr..

[48] Mukhopadhyay A, Bhatla N, Kriplani A, Agarwal N, Saxena R (2004). Erythrocyte indices in pregnancy: effect of intermittent iron supplementation. Natl Med J India.

[49] Mukhopadhyay A, Bhatla N, Kriplani A, Pandey RM, Saxena R (2004). Research G: Daily versus intermittent iron supplementation in pregnant women: hematological and pregnancy outcome. J Obstetr Gynaecol Res..

[50] Sharma JB, Jain S, Mallika V, Singh T, Kumar A, Arora R, Murthy NS (2004). A prospective, partially randomized study of pregnancy outcomes and hematologic responses to oral and intramuscular iron treatment in moderately anemic pregnant women. Am J Clin Nutr..

[51] Kumar A, Jain S, Singh N, Singh T (2005). Obstetrics: Oral versus high dose parenteral iron supplementation in pregnancy. Int J Gynaecol Obstet.

[52] Saha L, Pandhi P, Gopalan S, Malhotra S, Kumar Saha P (2007). Comparison of efficacy, tolerability, and cost of iron polymaltose complex with ferrous sulphate in the treatment of iron deficiency anemia in pregnant women. Med Gen Med..

[53] Bhutta ZA, Rizvi A, Raza F, Hotwani S, Zaidi S, Hossain SM, Soofi S, Bhutta S (2009). A comparative evaluation of multiple micronutrient and iron–folic acid supplementation during pregnancy in Pakistan: impact on pregnancy outcomes. Food Nutr Bull..

[54] Wijaya-Erhardt M, Muslimatun S, Erhardt JG (2011). Fermented soyabean and vitamin C-rich fruit: a possibility to circumvent the further decrease of iron status among iron-deficient pregnant women in Indonesia. Public Health Nutr..

[55] Choudhury N, Aimone A, Hyder SZ, Zlotkin SH (2012). Relative efficacy of micronutrient powders versus iron—folic acid tablets in controlling anemia in women in the second trimester of pregnancy. Food Nutr Bull..

[56] Magon A, Collin SM, Joshi P, Davys G, Attlee A, Mathur B (2014). Leaf concentrate fortification of antenatal protein-calorie snacks improves pregnancy outcomes. J Health Popul Nutr..

[57] Kamdi S, Palkar PJ (2015). Efficacy and safety of ferrous asparto glycinate in the management of iron deficiency anaemia in pregnant women. J Obstet Gynaecol..

[58] Mehta R, Platt AC, Sun X, Desai M, Clements D, Turner EL (2017). Efficacy of iron-supplement bars to reduce anemia in urban Indian women: a cluster-randomized controlled trial. Am J Clin Nutr..

[59] Jose A, Mahey R, Sharma JB, Bhatla N, Saxena R, Kalaivani M, Kriplani A (2019). Comparison of ferric Carboxymaltose and iron sucrose complex for treatment of iron deficiency anemia in pregnancy-randomised controlled trial. BMC Pregnancy Childbirth..

[60] Ahamed F, Yadav K, Kant S, Saxena R, Bairwa M (2018). Pandav CSJIjoph: Effect of directly observed oral iron supplementation during pregnancy on iron status in a rural population in Haryana. Indian J Public Health.

[61] Lipiński P, Styś A, Starzyński RR (2013). Molecular insights into the regulation of iron metabolism during the prenatal and early postnatal periods. Cell Mol Life Sci.

[62] Andrews NC, Schmidt PJ (2007). Iron homeostasis. Annu Rev Physiol.

[63] Andrews NC (2000). Iron metabolism: iron deficiency and iron overload. Annu Rev Genomics Hum Genet.

[64] Ems T, St Lucia K, Huecker MR: Biochemistry, Iron Absorption. In: StatPearls. edn. Treasure Island (FL): StatPearls Publishing. Copyright © 2021, StatPearls Publishing LLC.; 2021. 28846259

[65] Thankachan P, Walczyk T, Muthayya S, Kurpad AV, Hurrell RF (2008). Iron absorption in young Indian women: the interaction of iron status with the influence of tea and ascorbic acid. Am J Clin Nutr.

[66] Hurrell R, Egli I (2010). Iron bioavailability and dietary reference values. Am J Clin Nutr.

[67] Unger HW, Bleicher A, Ome-Kaius M, Aitken EH, Rogerson SJ (2022). Associations of maternal iron deficiency with malaria infection in a cohort of pregnant Papua New Guinean women. Malar J.

[68] Singal N, Setia G, Taneja BK, Singal KK (2018). Factors associated with maternal anaemia among pregnant women in rural India. Bangladesh J Med Sci.

[69] Ramakrishnan U, Grant F, Goldenberg T, Zongrone A, Martorell R (2012). Effect of women's nutrition before and during early pregnancy on maternal and infant outcomes: a systematic review. Paediatr Perinat Epidemiol.

[70] Bhutta ZA, Das JK, Rizvi A, Gaffey MF, Walker N, Horton S, Webb P, Lartey A, Black RE (2013). Evidence-based interventions for improvement of maternal and child nutrition: what can be done and at what cost?. Lancet.

[71] Rabindrakumar MSK, Wickramasinghe VP, Gooneratne L, Arambepola C, Senanayake H, Thoradeniya T (2018). The role of haematological indices in predicting early iron deficiency among pregnant women in an urban area of Sri Lanka. BMC Hematol..

[72] Lopez A, Cacoub P, Macdougall IC, Peyrin-Biroulet LJTL (2016). Iron deficiency anaemia.

